# Mutual regulations between *Toxoplasma gondii* and type I interferon

**DOI:** 10.3389/fimmu.2024.1428232

**Published:** 2024-07-08

**Authors:** Lingling Song, Ruoyu Wang, Yuanyuan Cao, Li Yu

**Affiliations:** ^1^ Department of Microbiology and Parasitology, Anhui Province Laboratory of Zoonoses, School of Basic Medical Sciences, Anhui Medical University, Hefei, Anhui, China; ^2^ The Rausser College of Natural Resources, University of California, Berkeley, CA, United States

**Keywords:** T.gondii, IFN-I, innate immunity, immune evasion, treatment

## Abstract

In the decades since the discovery, Type I interferon (IFN-I) has been intensively studied for their antiviral activity. However, increasing evidences suggest that it may also play an important role in the infection of *Toxoplasma gondii*, a model organism for intracellular parasites. Recent studies demonstrated that the induction of IFN-I by the parasite depends on cell type, strain genotype, and mouse strain. IFN-I can inhibit the proliferation of *T. gondii*, but few studies showed that it is beneficial to the growth of the parasite. Meanwhile, *T. gondii* also can secrete proteins that impact the pathway of IFN-I production and downstream induced interferon-stimulated genes (ISGs) regulation, thereby escaping immune destruction by the host. This article reviews the major findings and progress in the production, function, and regulation of IFN-I during *T. gondii* infection, to thoroughly understand the innate immune mechanism of *T. gondii* infection, which provides a new target for subsequent intervention and treatment.

## Introduction

Interferon (IFN) was first identified and named in 1950s, when scientists found inactivated viruses lead cells to produce a substance to inhibit the replication of the viruses (1, 2). There are three types of IFNs, namely type I interferon (IFN-I), type II interferon (IFN-II), and type III interferon (IFN-III). IFN-I shows the greatest diversity, with over 20 family members, mainly including IFN-β and subtypes of IFN-α. Almost all cells can produce IFN-I, in which IFN-α is mainly produced by leukocyte and plasmacytoid dendritic cells (pDCs), and IFN-β is mainly produced by fibroblasts. IFN-II consists of IFN-γ, which is mainly produced by activated T cells and natural killer cells. IFN-III is also known as IFN-λ with four subtypes, including IFN-λ1, IFN-λ2, IFNλ3, and IFN-λ (3). *Toxoplasma gondii* is a model organism for intracellular parasites. The infection caused by *T. gondii* frequently presents itself in a latent form, typically without eliciting notable clinical manifestations. However, in individuals with compromised immune systems who are infected with *T. gondii*, the dormant parasites within the body can be reactivated (4, 5). This reactivation process can have deleterious effects on various organs, potentially extending to the development of mental health conditions. Of particular concern is the infection of *T. gondii* during the initial stages of pregnancy, as it can result in vertical transmission to the fetus. This infection can severely impact the fetus, possibly leading to miscarriage, premature birth, fetal abnormalities, and intellectual disabilities (6). The threat posed by toxoplasmosis to human health underscores the crucial importance of research endeavors aimed at its prevention and management. Although IFN-II (IFN-γ) has long been recognized as an essential anti-*T. gondii* immune effector, much less is known about the role of IFN-I in host defense against this parasite. Since its first discovery, IFN-I has been intensively studied for decades for their antiviral activity. However, increasing evidence suggests that IFN-I also plays an important role in limiting the infection of *T. gondii*, particularly in chronic infections which take place in brain (7). Meanwhile, when facing the deadly killing of the IFN-I, *T. gondii* secretes a type of proteins that hijack the host immunity to assist in successful immune escape (8–10). The balance between eliminating and escaping leads *T. gondii* building long-term infection. Interferons are crucial for orchestrating the immune response that limits the parasite’s growth. However, *T. gondii* has developed sophisticated mechanisms to combat the immune defenses mediated by these interferons, enabling it to evade the host’s immune surveillance and persist within the host cells (11). More and more studies show that there are mutual regulations between IFN-I and *T. gondii*. In this review, we will discuss the interaction between *T. gondii* and IFN-I, summarize and clarify the mechanism of innate immunity activated by IFN-I to counter *T. gondii*, and how the parasites evade immune killing by IFN-I.

## Signaling pathways for IFN-I production and function

### Signaling pathways for IFN-I production

**Figure 1:**
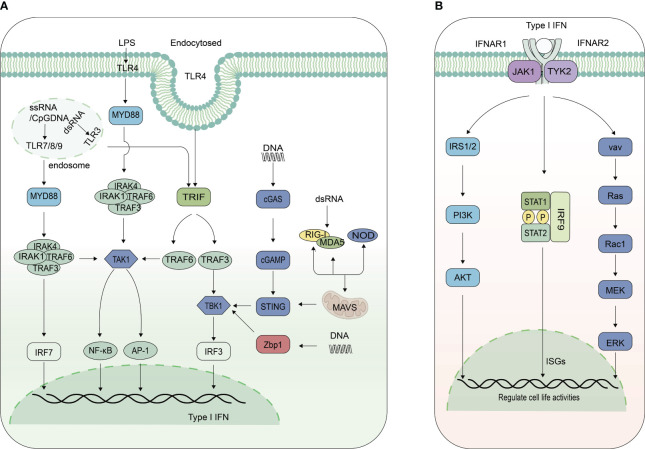
Signaling pathways for IFN-I production and function. **(A)** Signaling pathways inducing IFN-I expression. Toll-like receptor 7/8/9 (TLR7/8/9) predominantly activate the myeloid differentiation primary response 88 (MyD88)-TNF receptor-associated factor 6 (TRAF6)-IRF7-IFN-I pathway, whereas TLR3 triggers the TIR-domain-containing adapter-inducing interferon-β (TRIF)-TRAF3-IRF3 pathway, and TLR4 activates both. DNA is recognized by cyclic GMP-AMP synthase (cGAS) and Z-DNA binding protein 1 (Zbp1), subsequently activating the TANK-binding kinase 1(TBK1)-IRF3-IFN-I pathway. Phosphorylated IRF7 or IRF3, in conjunction with activated nuclear factor kappa-light-chain-enhancer of activated B cells (NF-κB) and activator protein 1 (AP-1), induces type I interferon production. Retinoic acid-inducible gene I (RIG-I) and melanoma differentiation-associated protein 5 (MDA5) recruit adaptor protein mitochondrial antiviral signaling protein (MAVS), which recruits TRAF3, forming a complex with TBK1 and IRF3, then initiate the production of IFN-I. Nucleotide-binding oligomerization domain-containing proteins (NOD) also modulate IFN-I production. **(B)** Signaling pathways for IFN-I function. IFN-I binds to Interferon alpha and beta receptor subunits 1 and 2 (IFNR1/IFNR2), activating janus kinase 1 (JAK1) and tyrosine kinase 2 (TYK2), which leads to the phosphorylation of signal transducer and activator of transcription 1 and 2(STAT1 and STAT2), forming heterodimers. These heterodimers, together with IRF9, constitute the interferon-stimulated gene factor 3 (ISGF3) complex. This complex is then translocated into the nucleus, where it binds to the interferon-stimulated response element (ISRE) sites of interferon-stimulated genes (ISGs), thereby promoting their expression. IFN-I also interacts with its receptor to activate mitogen-activated protein kinase (MAPK) and phosphatidylinositol 3-kinase (PI3K) pathways, regulating cellular activities.

The mammalian innate immune system has evolved various pattern recognition receptors (PRRs) to detect pathogens and damage-associated molecular patterns (PAMPs and DAMPs) to induce the production of IFN-I, activating the host innate immunity rapidly. Toll-like receptors (TLRs) are the most well studied PRRs. Eleven TLRs have been identified in humans and at least 13 were identified in mice. The Leucine-rich repeat (LRR) domain of TLRs is mainly responsible for the recognition of PAMPs, and the Toll/interleukin-1 receptor (TIR) domain is responsible for the recruitment of downstream TIR-containing adaptor proteins (12). TLR7, TLR8, and TLR9 are highly expressed in DCs. TLR7 and TLR8 mainly recognize single-strand RNA (ssRNA) and a few short double-strand RNA (dsRNA), while TLR9 mainly senses unmethylated DNA containing CpG motifs. They trigger IFN-I production through the myeloid differentiation factor-88 (MYD88)-dependent pathway, in which TNF receptor-associated factor 6 (TRAF6) is recruited by MYD88 and then forms a complex with interferon regulatory factor 7 (IRF7), thus phosphorylating IRF7. Phosphorylated IRF7 induces IFN-I production together with activated nuclear factor kappa-light-chain-enhancer of activated B cells (NF-κB) and activator protein 1 (AP-1) (13, 14). TLR3 mainly recognizes dsRNA in endosome, subsequently recruits TIR domain-containing adaptor (TRIF), finally activating TRIF-TNF receptor-associated factor 3 (TRAF3)-TANK-binding kinase 1 (TBK1)-interferon regulatory factor 3 (IRF3) pathway (**Figure 1A**) (15–17). TLR4 can be activated by LPS at the membrane surface to induce inflammatory cytokines through MYD88-dependent. Endocytosed TLR4 can be responsible for the induction of IFN-I and inflammatory cytokines in a TRIF dependent manner (**Figure 1A**) (18).

RIG-I-like receptors (RLRs) and melanoma differentiation-associated gene 5 (MDA5) have a caspase activation and recruitment domain (CARD) on their N terminal, with an RNA helicase domain in the middle. RIG-I recognizes 5-triphosphatedsingle-stranded RNA (5’ppp-ssRNA) and short dsRNAs, while MDA5 recognizes long dsRNAs. After combined with PAMPs, RIG-I and MDA5 recruit adaptor protein MAVS using the CARD (19). MAVS has similar sequence like RIG-I and MDA5 containing CARD. Subsequently, it recruits TRAF3, forming a complex with TBK1 and IRF3, then initiate the production of IFN-I (**Figure 1A**) (20).

Other PRRs, such as the Nod-like receptor (NLR) family also regulate IFN-I production. The NLR family has 23 proteins in humans and 34 proteins in mice (21). The structure of this family is characterized by variable N-terminus and rich leucine repeats in C-terminus, with nucleotide binding domain (NBD) in the middle. The NLR are mainly responsible for pro-inflammatory factors production activation such as IL-1 and IL-18 through the NF-κB pathway. NOD-containing protein1 (NOD1) and NOD2 can positively regulate the production of IFN-I. NOD1 and NOD2 regulate the production of IFN-β, depending on receptor-interacting serine/threonine-protein kinase 2 (RIPK2) (22, 23). NOD1 can target MDA5 and TRAF3 to form MDA5-MAVS and TRAF3-MAVS complex, thereby up-regulating the expression of IFN-I and ISGs (24). NOD2 also regulate the phosphorylation of IRF5 to induce the expression of IFN-I (**Figure 1A**) (23).

Cyclic GMP-AMP synthase (cGAS) has been identified as the main DNA sensor that can generate the second messenger 2’3’-cGAMP upon detection of cytosolic DNA. Then, stimulator of interferon genes (STING) on the endoplasmic reticulum (ER) binds with 2’3’-cGAMP, and the complex is transferred from ER to the Golgi, leading to the activation of TANK-binding kinase 1 (TBK1) and IRF3 for IFN-I and inflammatory cytokine production (25, 26). cGAS primarily recognizes DNA from pathogenic microorganisms associated with infection, but can also recognize endogenous DNA, including from nuclear and mitochondrial DNA. In addition to inducing the secretion of IFN-I, autophagy and lysosomal dependent cell death can also be induced by cGAS-STING pathway (27). Z-conformation acid binding protein 1 (ZBP1) is also a DNA sensor, which lies between two N-terminal Z-DNA binding domains (ZBD, called Zα1 and Zα2) to bind with DNA, two intermediate RIP homotypic interaction motif (RHIM, RHIM1 and RHIM2), and a C-terminal signal domain (SD). C-terminal signal domain is the main functional domain to induce IFN-I, which can recruit and activate TBK1 and IRF3 to promote the production of IFN-I (**Figure 1A**) (28, 29).

### Signaling pathways for IFN-I function

All IFN-I signal through a shared heterodimeric receptor, IFNAR, which is comprised of IFNAR1 and IFNAR2 subunits, to activate Janus kinase (JAK)- signal transducer and activator of transcription (STAT) signal pathway. JAK kinases, including JAK1, JAK2, JAK3 and Tyk2, have Src homology domain (SH2) kinase activity, which can phosphorylate the cytokine receptor (30, 31). The STAT family include STAT1, STAT2, STAT3, STAT4, STAT5a, STAT5b, and STAT6. They consist of SH2 domain and nuclear localization signal in the N-terminal, with DNA domain in the middle, and C-terminal Tyr domain. IFN-I acts as a ligand to bind to IFNAR1 and IFNAR2 on the cell surface, causing receptor polymerization, activating receptor-coupled kinases JAK1 and Tyk2, followed by the recruitment and phosphorylation of STAT1 and STAT2. Phosphorylated STAT forms a heterodimer, which together with IRF9 form interferon stimulated gene 3 (ISGF3) complex (**Figure 1B**). The nuclear localization signal was exposed and then transport into the nucleus, to bind to the interferon stimulated response elements (ISRES) of ISGs. Ultimately, ISGs are transcribed and translated to fight against viruses and bacteria (**Figure 1B**).

In addition to JAK-STAT pathway, IFN-I also binds to the receptor to activate mitogen activated protein kinases (MAPK) and phosphoinositide 3-kinase (PI3K) pathway. IFN-I activates downstream TYK2, which induces tyrosine phosphorylation of insulin receptor substrate 1 (IRS1) and IRS2, and then recruits PI3K to further activate the serine/threonine kinase AKT, which in turn activates or restrains downstream proteins to regulate cell activity (32, 33). TYK2 is also able to activate the guanosine nucleotide exchange factors vav, which subsequently activates small G proteins RAS and Rac1, ultimately leading to activation of downstream MAPK kinases and downstream signaling cascades (34). The two pathways play an important role in cell proliferation, differentiation, apoptosis etc (**Figure 1B**) (35, 36).

## Regulations between IFN-I and *T. gondii*

### The subtle role of IFN-I in *T. gondii* infection

In contrast to the well understood role of IFN-II in control of toxoplasmosis, the role of IFN-I is less clear. Early in the 1980s, scientists screened out the cytokines that may be effective against *T. gondii.* In macrophages, they screened IFN-α, IFN-β, colony-stimulating factor-1 (CSF-1), granulocyte and macrophage colony stimulating factor (GM-CSF), pluripotent colony stimulating factor (p-CSF), and migration inhibitory factor (MIF), and the results showed that IFN-α and IFN-β, with concentration of 300–500U/ml, had no anti- *T. gondii* activity in human macrophages (37). Similarly, IFN-α and IFN-β failed to induce antimicrobial activity, though they stimulated the expression of indoleamine 2,3-dioxygenase (IDO) in human monocyte-derived macrophages (38). However, research showed that recombinant IFN-I (300–3000U/ml) inhibited survival and replication of *T. gondii* in human adults, neonates monocyte, and WISH cells, despite being less effective than IFN-γ (**Table 1**) (39). Moreover, previous study demonstrated that the recombinant IFN-β and LPS would inhibit the growth of *T. gondii* in monocytes through IDO induction. In this process, exogenous tryptophan reversed this inhibitory effect (46). Also, in human retinal pigment epithelium (HRPE) cell, IFN-I was found to inhibit *T. gondii* replication in a dose and IDO dependent manner but independent on nitric oxide (NO) (40). Recent studies showed that IFN-β inhibited rosette formation and wiped out the PLK strain in murine macrophages and fibroblasts by increasing immunity-related GTPase M1 (IRGM1) around the parasitophorous vacuole (PV) membrane, independent from IDO and inducible nitric oxide synthase (iNOS) (**Table 1**) (41). These observed differences in IFN-I antiparasitic actions *in vitro* by various investigators could be due to the inherent properties of the types of cells, concentration, purity of recombinant IFN-I, and sensitivities of *T. gondii* proliferation detection methods used in each study.

**Table 1:** The roles of IFN-I in *T. gondii* infection.

*In vitro/* *In vivo*	Host/ Cell type	Parasite strain (Type)	Results	Mechanism	Reference
*In vitro*	Human macrophages	Unknown	No anti *T. gondii* activity with 300–500U/ml	Cannot enhance the H2O2 secretion	(37)
*In vitro*	Human monocyte-derived macrophages, WISH	Unknown	Recombinant IFN-I inhibited survival and replication of *T. gondii* with less effective than IFN-γ	Undetermined	(39)
*In vitro*	HRPE	RH (Type I)	IFN-I inhibits *T. gondii* replication	NO-independent but IDO-dependent mechanisms (Nagineni et al.,1996)	(40)
*In vitro*	Murine macrophages/fibroblasts	PLK (Type II)	IFN-β contributes in eliminating T. gondii	Recruitment of IRGM1 around the PV membrane.	(41)
*In vivo*	Rabbits	RH (Type I)	IFN inducer Poly(I:C) delayed the production of ocular lesions	Undetermined	(42)
*In vivo*	C57 BALB/c	RH (Type I)	IFN-I plays a protective role in experimental ocular toxoplasmosis	The eye tissue of BALB/c mice produce much higher levels of IFN than C57	(43)
*In vivo*	ifnαr1-/-C57	ME49 (Type II)	IFN-I controls *In vivo* expansion of acute and chronic T. gondii infection	TgIST blocks IFN-I signaling	(9)
*In vivo*	C57 ifnαr-/- C57	PRU (Type II)	IFN-I is harmful to host anti-toxoplasmosis immunity	IFN-I deteriorates host anti-*toxoplasma* immunity via dampening T-cell function	(44)
*In vivo*	ifnαr-/- C57	PRU (Type II)	IFN-I protects from *T. gondii* infection	Undetermined	(45)

*In vivo* infection model, an IFN inducer, polyinosine-polycytidylic acid (Poly I:C), was first experimented in male New Zealand white rabbits one day before RH strain of *T. gondii* (Type I) infection. Resulting data showed that Poly I:C delayed the production of ocular lesions caused by the parasite (42). In mice infection model, toxoplasmosis caused by RH in C57BL/6 mice was found to be more severe than in BABL/c mice, and the severity of the disease negatively correlated with the level of IFN-I (43). This suggests that IFN-I may have a protective role in toxoplasmosis. Gene knockout mice are an important model for studying gene function as well. To further get into the details of IFN-I in toxoplasmosis, mice lacking IFN-I receptors (*Ifnαr1^-/-^*) were applied to many studies. *Ifnαr1* ^-/-^ mice on C57BL/6 background showed significantly higher susceptibility to oral challenge test using tissue cysts of ME49 (type II) parasites when compared with wild-type (WT) mice. Moreover, the *Ifnαr1* ^-/-^ mice showed a significantly higher cyst burden and more severe pathological changes in brain than the WT mic (9). This indicates that IFN-I is doing well in protecting mice from *T. gondii* infection. However, conflicting conclusions have been drawn regarding the role of IFN-I in *T. gondii* infection when using PRU parasites. An early study showed that *Ifnαr1* ^-/-^mice fed with PRU cysts had higher parasite loads and reduced survival. The results suggested that IFN-I provide certain protective mechanism in mice (**Table 1**) (45). By contrast, *Ifnαr1^-/-^* mice intraperitoneally infected with PRU tachyzoites showed increased survival when compared with WT mice. Exogenous supplement of IFN-β in WT mice resulted in severe weight loss, more parasite loads, and higher mortalities within 9 days post infection. At the same time, exogenous IFN-β was found to promote the proliferation of PRU in L929 cells. Researchers further proved that inflammasomes could provide protection by blocking the IFN-I signaling pathway (**Table 1**) (44). The differences in these results may be due to differences in genotype, dose, and route of infection. Although exogenous supplement of IFN-β in WT mice resulted in higher mortalities, this does not indicate that IFN-I does not have protective effect in *T. gondii* infection. It has been previously reported that IFN-β treatment after infection increased lethality because of driving inflammatory cell death and lethality (47). IFN-I seems to be a double-edged sword in *T. gondii* infection. It is still unknown which factors determine IFN-I as host-protective or pathogenic.

IFN-I initiates the JAK-STAT signaling cascade through binding to its homologous receptors, subsequently inducing the expression of numerous ISGs. Many ISGs have been reported to exert significant effects on *T. gondii*, such as GBPs, interferon-stimulated gene 15 (ISG15), PKR (protein kinase R), and transporter associated with antigen processing 21(TRAM21). GBPs are recruited to the PV membrane, leading to the destruction of the PV (48, 49). ISG15 has been demonstrated an ability to promote the release of IFN-γ and IL-1β during the early infection of *T. gondii*. Additionally, it functions as a bridging molecule between autophagy and IFN-γ signaling, thereby aiding in the limitation of *T. gondii* proliferation (50, 51). PKR can stimulate the autophagy pathway, resulting in the elimination of *T. gondii* (52). Furthermore, research indicates that TRIM21 localizes to the *T. gondii* type II vacuole and plays a crucial role in cytokine production and parasite restriction during *T. gondii* infection (53). These ISGs may act as candidate effector molecules for IFN-I mediated resistance against Toxoplasma gondii. Apart from the aforementioned mechanisms, IFN-I can also induce the secretion of various cytokines and chemokines. Previous studies have shown that IFN-I has the capacity to recruit NK cells and trigger their release of IFN-γ (54). During *T. gondii* infection, IFN-I facilitates the recruitment of inflammatory cells and augments the secretion of IFN-γ by NK cells. Concurrently, both Type I and III interferons can trigger the release of chemokines and cytokines during a *T. gondii* infection, while positively modulating zonula occluden-1 (ZO-1). This modulation contributes to maintaining barrier integrity and potentially restricts the dissemination of *T. gondii* between tissues (55).

In summary, IFN-I exerts their antiparasitic effects against *T. gondii* primarily through several mechanisms. Firstly, they create an intracellular environment that is hostile to the proliferation of the parasite by augmenting the production of molecules such as NO and IDO, as well as by modulating the metabolic pathways within the host cell. Additionally, IFN-I stimulates the expression of a range of downstream ISGs that directly impede the replication and growth of *T. gondii*. Furthermore, IFN-I bolsters the immune response by promoting the secretion of various cytokines, thereby orchestrating a coordinated defense against the parasite. Concurrently, IFN-I modulates the recruitment and activity of inflammatory cells, further aiding in the suppression of *T. gondii* proliferation. While studies investigating the role of IFN-I in *T. gondii* infection still harbor certain limitations, accumulating evidence suggests that IFN-I possesses protective mechanisms in both chronic and acute infections. However, the specific mechanism of IFN-I in *T. gondii* infection still needs to be further elucidated by establishing different infection models utilizing a wider range of parasite strain genotypes.

### *T. gondii* infection affects the expression of IFN-I

Early in the 1960s, it was found that the mice infected with *T. gondii* produced an interferon-like substance to inhibit replication of vesicular stomatitis virus (VSV) and Mengo virus (56, 57). Subsequently, in the 1980s, researchers found that when mice were stimulated with *T. gondii* lysate antigen (TLA) from RH, the level of IFN-I showed different correlations with different strains of mice. C57BL/6 was the best strain of interferon production, followed by A/J, DBA/2, C3H/He, and BALB/c is the least one to produce IFN-I. Compared with BALB/c, the survival time of C57BL/6 was extended following primary or secondary infection with *T. gondii* (58). It is speculated that the sensitivity of different strains of mice to *T. gondii* is related to its ability to produce IFN-I. In a model of acute ocular *T. gondii* infection, experiments had shown that the eye tissue of BALB/c mice produced much higher levels of IFN-I than C57BL/6, and C57BL/6 showed more severe eye lesions and higher parasitic load in the eye (43). This result suggests that the production of IFN-I is related to the strain of the mouse and IFN-I seems to protect the body from *T. gondii*. The differences in the above results demonstrate the differences in administrations in mice (TLA or live tachyzoites) and test specimens (serum or eye tissue). Blood samples reflect the immune system of the whole body, while eye tissue only reflects local immune. When *T. gondii* infect mice, at what point does interferon expression begin to increase? About 24h after C57BL/6 by PRU infection, serum IFN-β began to increase expression. While the levels of IFN-β in the peritoneal cells increased significantly 6 days after infection (dpi) in C57BL/6 peritoneally infected with ME49 tachyzoites. The levels of IFN-I in the brain of BABL/c mice infected with PLK strain started to rise at 6 dpi, and in spleen increased at 8–10 dpi (9, 41, 44). These results indicate that the expression of IFN-I begins to increase at the early stage of infection, and may play an important role during the early stage of infection.

**Figure 2:**
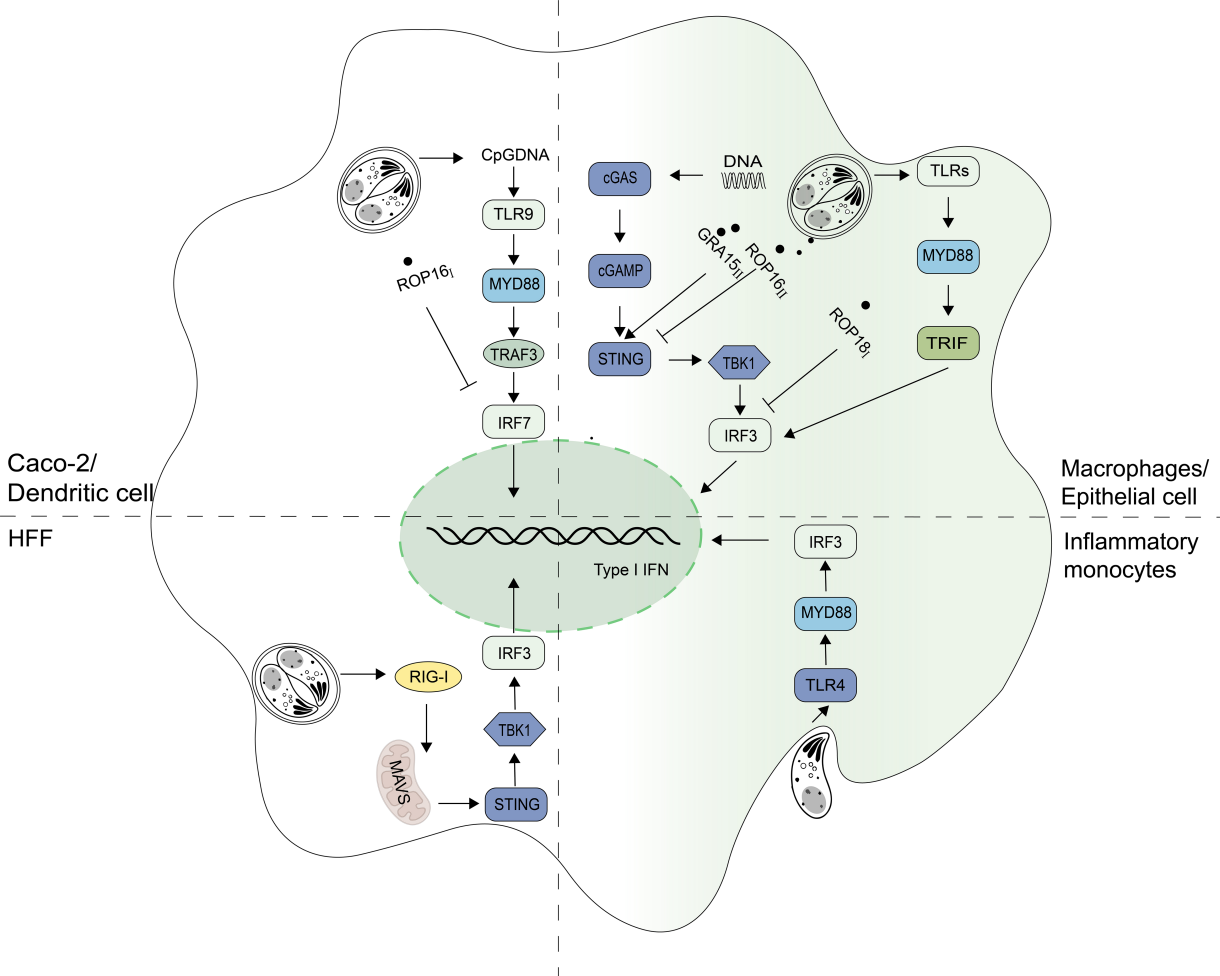
Signaling pathways for IFN-I regulation in *T. gondii* infection. Signaling pathways inducing Type I interferon (IFN-I) expression depend on cell types during T. gondii infection. Unmethylated CpG DNA from RH strains can increase IFN-I levels via the toll-like receptor 9 (TLR9) pathway in Caco-2 cells. In macrophages, *T. gondii* activates the cyclic GMP-AMP synthase (cGAS) - stimulator of interferon genes (STING) pathway and relies on the TLRs-myeloid differentiation primary response 88 (MYD88) pathway to produce IFN-I. The retinoic acid-inducible gene I (RIG-I) - mitochondrial antiviral-signaling protein (MAVS) pathway is mainly dependent in fibroblasts. When *T. gondii* is in inflammatory monocytes, the TLR4-MYD88 pathway is primarily relied upon to produce IFN-I. GRA15*_II_* promotes STING polyubiquitination and thereby enhances the host DNA/cGAS/STING pathway against *T. gondii* infection. ROP18*_I_* binds to IRF3 and inhibits the TANK-binding kinase 1 (TBK1)- Interferon regulatory factor 3 (IRF3)-IFN-β pathway. ROP16*_I_* phosphorylates signal transducers and activators of transcription 3 (STAT3) and inhibits nuclear translocation of Interferon regulatory factor 7 (IRF7), blocking IFN-α production. ROP16*_II_* can also inhibit innate immunity by targeting the cGAS-STING pathway.

In addition to *in vivo* infection, *T. gondii* infection *in vitro* can also affect IFN-I production (**Table 2**). A previous study showed that the production of IFN-I increased when RAW.264.7 infected with RH (8). In mouse pDCs, the infection with PRU also induced the production of IFN-α 48h after infection (59). In addition to the cell type, genotype of *T. gondii* is also considered to be the reason for the difference in its ability to induce IFN-I. Transcriptomic data showed that most types of strains could not cause IFN-I production, and only a few atypical strains like COUGAR could (60). when *T. gondii* invades host cells, the host induces IFN-I through different signal pathways by recognizing different molecular patterns of the pathogen. It was reported that it depends not only on RIG-I pathways in mouse fibroblasts but also MYD88/TRIF pathways in mouse macrophages (**Figure 2**, **Table 2**) (60). Research have shown that the induction of IFN-I by *T. gondii* is related to the mode of *T. gondii* entering cells and the state of *T. gondii*. It is important to note that inflammatory monocytes (IMs), not neutrophils, were found to produce IFN-β in response to *T. gondii*. Moreover, researchers found that IFN-β production requires phagocytic uptake of *T. gondii* by the host. IFN-β production is reduced in hosts lacking TLR4 or MyD88 (**Figure 2**, **Table 2**) (45). Unmethylated CpG DNA purified from RH strain can up-regulate TLR9 in Caco-2 cells and induce DEFA-5 secretion through the release of cytokine IFN-β (**Figure 2**) (61). In addition to an increase of IFN-I production, *T. gondii* infection has also been found to inhibit IFN-I production. In HFF cells, live *T. gondii* did not elicit IFN-I response while heat-inactivated *T. gondii* boosted the IFN-β production. Furthermore, when co-infected with VSV-GFP, the IFN-β production was impaired by an unknown soluble substance released by *T. gondii* (62). In pDCs, *T. gondii* infection was found to inhibit nuclear translocation of IRF7, induced by viral infection, thereby reducing IFN-α production when co-infected with herpes simplex virus (HSV) or human immunodeficiency virus (HIV) (63).

**Table 2:** The expression of IFN-I induced by T. gondii.

*In vitro/* *In vivo*	Host /Cell type	Parasite strain (Type)	Results	Mechanism	References
*In vitro*	Mouse pDCs	PRU (Type II)	High levels of IFN- α	TLR11-dependent fashion	(59)
*In vitro*	Mouse fibroblasts/macrophages /HFF	COUGAR (Atypical)	Induce strong IFN-β signature	TLRs dependent in mouse macrophages, and RIG-I pathways in mouse fibroblasts	(60)
*In vitro*	Transfected Caco-2 cells	RH Unmethylated CpG DNA (Type I)	Induce IFN-β secretion	Activate TLR9/IFNβ/DEFA-5 pathway	(61)
*In vitro*	Mouse monocytes	PRU (Type II)	Induce IFN-β secretion	Phagocytic uptake of *T. gondii* to trigger IFN-β through TLR4/MyD88	(45)
*In vitro*	RAW.264.7	RH (Type I)	The production of IFN-I is increased	Undetermined	(8)
*In vitro*	HFF	Heat-killed RH (Type I)	Induction of the IFN-I	Recognized by innate receptors through internalized	(62)
*In vitro*	HFF	RH (Type I)	RH inhibits VSV-induced IFN-I levels	Suppress the IFN-I pathway with unknown substance	(62)
*In vitro*	Human pDC	RH (Type I)	Suppress VSV-induced IFN-I levels	ROP16 inhibits nuclear translocation of IRF7	(63)
*In vivo*	Swiss-Webster mice	ME49/RH (Type II/I)	Produces interferon like substance	Undetermined	(57)
*In vivo*	C57 BALB/c	TLA from RH (Type I)	IFN-I highest in C57BL/6, and lowest in BALB/c	Undetermined	(58)
*In vivo*	C57 BALB/c	RH (Type I)	Eye tissue of BALB/c produce higher levels of IFN-I than C57BL/6	Undetermined	(43)
*In vivo*	C57	PRU (Type II)	Serum IFN-β increased at 24h after infection	Enhanced Phosphorylation of TBK1	(44)
*In vivo*	C57	ME49 tachyzoites (Type II)	At 6 dpi, the levels of IFN-β and IFN-a increased in the peritoneal cells	Undetermined	(9)
*In vivo*	BABL/c	PLK (Type II)	The levels of IFN-I in the brain and spleen start to rise in early stage	Undetermined	(41)

It is certain that *T. gondii* infection affects IFN-I expression. In *in vivo* and *in vitro* experiments, the production of IFN-I was influenced by various factors such as mouse strain type, *T. gondii* genotype, and cell type (**Table 2**). *T. gondii* not only promotes IFN-I production, but also secretes effector molecules that suppress IFN-I expression to aid in immune system invasion and survival rate. However, there is no consensus on how *T. gondii* infection affects host IFN-I levels, and the unknown soluble substance that inhibits IFN-I production worth further investigation.

### Counteractive strategies used by *T. gondii* against IFN-I

**Figure 3:**
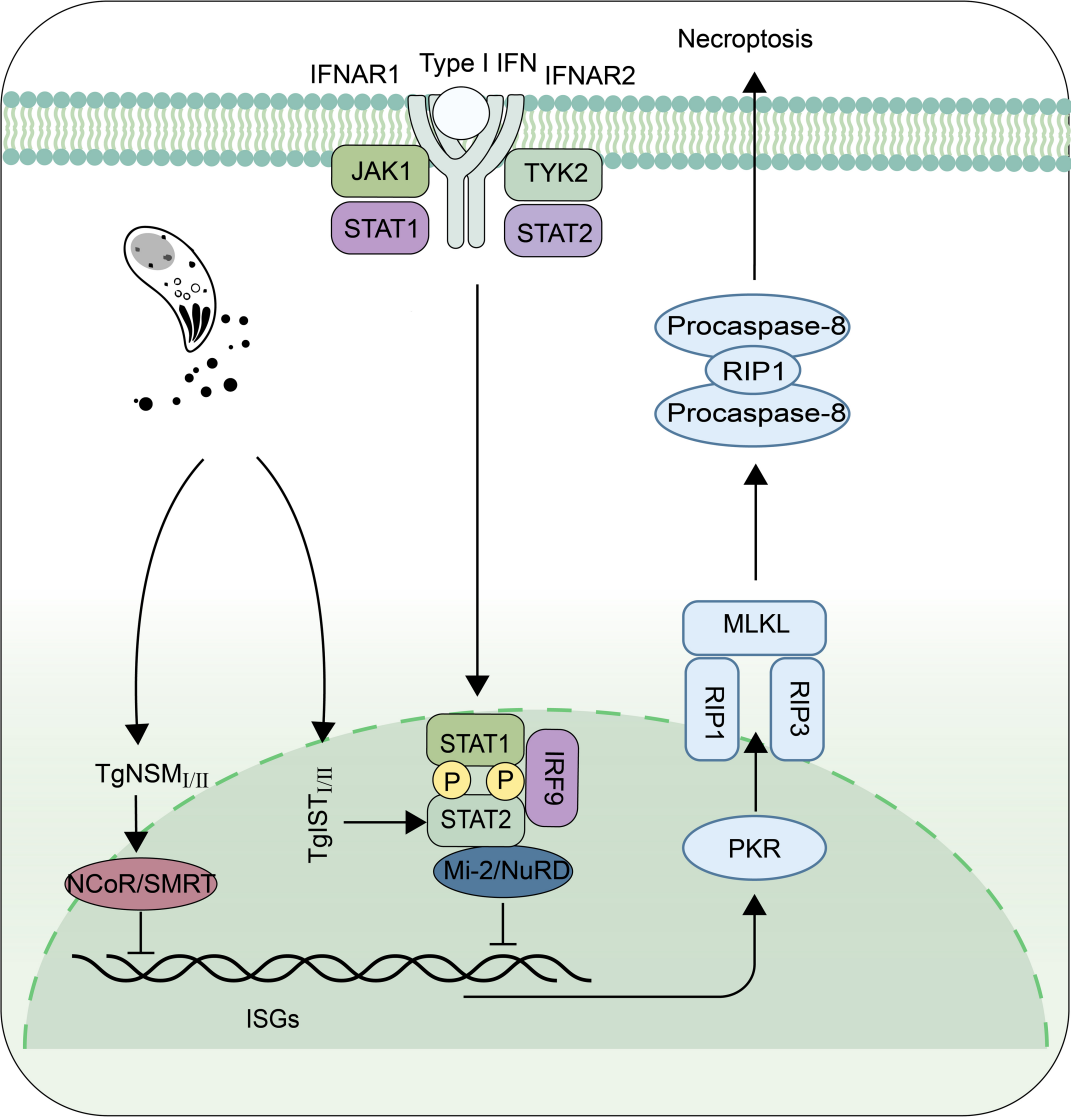
The proteins secreted by *T. gondii* resist the killing effect of Type I interferon. TgIST*_I/II_* and TgNSM*_I/II_* inhibit the IFN-β-activated necroptosis pathway by recruiting methyl-CpG binding protein 2/nucleosome remodeling and deacetylase (Mi-2/NuRD) and nuclear receptor co-repressor/silencing mediator of retinoic acid and thyroid hormone receptors (NCoR/SMRT) complexes, respectively. Protein Kinase R (PKR), Receptor Interacting Protein 1 (RIP1), Receptor Interacting Protein 3 (RIP3), Mixed Lineage Kinase L (MLKL).

Although the mechanisms of IFN-I on *T. gondii* have not been fully understood, many recent studies have shown that this parasite can regulate IFN-I signaling pathway through its different secreted proteins with genotype specificity, to enhance or escape from innate immune responses (**Figures 2**, **3**). Rhoptry protein 18 *_I_* (ROP18 *_I_ *), a well- known molecule that helps the parasite to evade from IFN-γ induced GTPase-mediated host defense system, was recently reported to inhibit the nuclear translocation of IRF3 by binding directly to IRF3 follows by the decrease of IFN-β expression both *in vivo* and *in vitro* (**Figure 2**) (8). These results partially explain the drastic decrease of RHΔROP18 virulence, compared with the wild-type RH strain. However, deletion of ROP18 *_I_* protein prevents immunity-related GTPases (IRGs) phosphorylation and disrupts IRGs localized to the PV membrane, which is the main reasons for the decreased virulence of RHΔROP18 strain (64, 65). ROP16 is a key virulence determinant and regulator of host cell transcription. It has kinase activity and can phosphorylate STAT3 and STAT6, causing down-regulation of the expression of some inflammatory cytokines (66). STAT3 has been found to negatively regulate IFN-I response (67, 68). ROP16 also induces macrophages shifting towards M2 direction (69). As mentioned earlier, in pDCs, when co-infected with virus, the ROP16*_I_* of RH can inhibit nuclear translocation of IRF7 to reduce virus-induced IFN-I (63). It is worth investigating whether ROP16*_I_* with kinase function phosphorylates other molecules of IFN-I signaling pathway. Whether other effector molecules of *T. gondii* also play a negative regulatory role in the stage of co-infection needs to be considered and further studied. Recently, research reported that ROP16*_II_* diminished IFN-I signaling, which independent on its kinase activity, via the inhibition of the cGAS pathway by suppressing K63-linked ubiquitination of STING (**Figure 2**) (70). However, the dense granule protein 15 (GRA15*_II_*), another protein from *T. gondii*, promotes STING polyubiquitination at Lys-337 and STING oligomerization through a TRAF protein–dependent process, and subsequently enhances the host DNA/cGAS/STING pathway against *T. gondii* infection (**Figure 2**) (71). The proteins secreted by *T. gondii* not only affect the signaling pathway of IFN-I production, but also the expression of ISGs. *T. gondii* inhibitor of STAT1-dependent transcription (TgIST) is conserved in the strains, and is a key virulence factor to the parasite. TgIST*_I/II_* was firstly found to block IFN-γ-dependent transcription. It has two distinct regions that function independently: a N-terminal region containing repeats bind directly to STAT1 dimers and a separate C-terminal region binds Mi-2/NuRD (72). However, a recent study has shown that TgIST*_I/II_* blocks IFN-I signaling to promote infection by binding to STAT1/STAT2 heterodimers and recruiting Mi-2 nucleosome remodeling and deacetylase (NuRD) complexes. Another possible mechanism could be that, TgIST*_I_* binds to the STAT1 dimer interface and induces a conformational change that inhibits the recruitment of co-transcriptional activators (9). This proposed mechanism may underpin the blocking of both type-I and type-II IFN production by infected host cells. The expression of IFN-I was up-regulated, and the survival time was prolonged in mice with PRUΔIST infection. *In vitro*, IFN-I could inhibit the growth of PRUΔIST in THP-1 and SH-SY5Y cells, and this inhibition was removed after complement with TgIST*_II_* (9). In addition, *T. gondii* NcoR/SMRT modulator (TgNSM*_I/II_*), a secreted effectors in chronic bradyzoite stage, was reported to target the silencing mediator of retinoic acid and thyroid hormone receptors (NCoR/SMRT) complex, a repressor for various transcription factors, to inhibit interferon-regulated genes involved in cell death. It acts with TgIST*_I/II_* to block IFN-driven expression of PKR and mixed-lineage-kinase-domain-like (MLKL), thus preventing host cell from necroptotic death (**Figure 3**) (10, 73). These diverse proteins originating from different strains of *T. gondii* exert varying effects on IFN-I, potentially explaining the differences in virulence among distinct strains of *T. gondii*.

## Summary

IFNs have been found to play various important roles in the regulation of bacterial, viral, and protozoal infections, and have been used as one of the treatment methods for human autoimmune diseases and infectious diseases. Unlike IFN-II, which has long been recognized as an essential effector in controling toxoplasmosis, less is known about the role of IFN-I in host defense against *T. gondii*. There is still a debate about whether IFN-I is beneficial or harmful to the growth of the parasite. Increasing evidences suggests that IFN-I plays an important role in limiting infection of *T. gondii*. However, more researches are needed to determine which IFN-I -inducible genes are important for cell intrinsic control of parasites. As one of the most successful parasites in the world, *T. gondii* develops a series of strategies to fight against host defense. To neutralize IFN-I signaling, it secretes ROP18 and ROP16 to inhibit the IFN-I production pathway, and produces TgIST and TgNSM at the same time to deactivate ISGs downstream of IFN-I, thus protecting *T. gondii* from host elimination. Many previous studies have shown that *T. gondii* infection triggers IFN-I production through DNA or RNA sensors. However, *T. gondii* is a unique intracellular pathogen different from viruses. It resides within a non-fusogenic PV, which provides a physical barrier for the parasite to shield from intracellular cytoplasmic defense mechanisms. It remains a mystery of how the host cell senses the DNA or RNA from the parasite or host to induce IFN-I production. In the future researches, we need to conduct in-depth research on these unknown questions mentioned above, which will provide theoretical basis and molecular means for the subsequent prevention and control of *T. gondii.*

## Author contributions

LS: Writing – original draft. RW: Writing – review & editing. YC: Writing – review & editing. LY: Writing – review & editing, Conceptualization, Supervision.
